# On the Efficacy of H_2_O_2_ or S_2_O_8_^2−^ at Promoting the Inactivation of a Consortium of Cyanobacteria and Bacteria in Algae-Laden Water

**DOI:** 10.3390/microorganisms10040735

**Published:** 2022-03-29

**Authors:** Javier Moreno-Andrés, Ignacio Rivas-Zaballos, Asunción Acevedo-Merino, Enrique Nebot

**Affiliations:** Department of Environmental Technologies, Faculty of Marine and Environmental Sciences, INMAR—Marine Research Institute, CEIMAR—International Campus of Excellence of the Sea, University of Cadiz, 11510 Puerto Real, Cádiz, Spain; ignacio.rivaszaballos@alum.uca.es (I.R.-Z.); asuncion.acevedo@uca.es (A.A.-M.); enrique.nebot@uca.es (E.N.)

**Keywords:** harmful algal bloom, hydrogen peroxide, persulfate, fenton, cyanobacteria, marine bacteria

## Abstract

Harmful algal blooms in coastal areas can significantly impact a water source. Microorganisms such as cyanobacteria and associated pathogenic bacteria may endanger an ecosystem and human health by causing significant eco-hazards. This study assesses the efficacy of two different reagents, H_2_O_2_ and S_2_O_8_^2−^, as (pre-)treatment options for algae-laden waters. *Anabaena* sp. and *Vibrio alginolyticus* have been selected as target microorganisms. With the objective of activating H_2_O_2_ or S_2_O_8_^2−^, additional experiments have been performed with the presence of small amounts of iron (18 µmol/L). For the cyanobacterial case, H_2_O_2_-based processes demonstrate greater efficiency over that of S_2_O_8_^2−^, as *Anabaena* sp. is particularly affected by H_2_O_2_, for which >90% of growth inhibition has been achieved with 0.088 mmol/L of H_2_O_2_ (at 72 h of exposure). The response of *Anabaena* sp. as a co-culture with *V. alginolyticus* implies the use of major H_2_O_2_ amounts for its inactivation (0.29 mmol/L of H_2_O_2_), while the effects of H_2_O_2_/Fe(II) suggests an improvement of ~60% compared to single H_2_O_2_. These H_2_O_2_ doses are not sufficient for preventing the regrowth of *V. alginolyticus* after 24 h. The effects of S_2_O_8_^2−^ (+ Fe(II)) are moderate, reaching maximum inhibition growth of ~50% for *Anabaena* sp. at seven days of exposure. Nevertheless, doses of 3 mmol/L of S_2_O_8_^2−^ can prevent the regrowth of *V. alginolyticus*. These findings have implications for the mitigation of HABs but also for the associated bacteria that threaten many coastal ecosystems.

## 1. Introduction

Harmful algal blooms (HABs) have become a global concern, especially during the last few years. The mechanisms that trigger an algal bloom episode are complex and are derived from natural processes, such as variation in temperatures that induce water stratification, to human-induced processes that may increase the number of nutrients released into waters. In this context, warm temperatures, as well as nutrient and light availability, are the basic requirements to sustain an extensive bloom [1]. When it is produced, wide impacts can be derived from such (micro)algal blooms, which could involve the eutrophication of waters or the release of associated toxins that will cause significant eco-hazards in both ecosystems and for human health [2,3,4].

Together with these blooms, the bacteria *Vibrio* spp. has also been reported, suggesting that the HABs may enhance the bacterial growth of these pathogenic species. This demonstrates a positive relationship between the abundance of *Vibrio* spp. and harmful phytoplankton, including both cyanobacterial or dinoflagellates bloom-related species [5,6]. *Vibrio* spp. are ubiquitously present in marine and estuarine environments, with fewer species being reported as pathogenic Vibrios for animals and plants. Approximately one dozen species have been known to cause infections in humans [7,8]. Specifically, there are particular pathogenic Vibrios that clearly dominate human infection; they are known as the “big four”: *V. cholerae, V. vulnificus, V. parahaemolyticus* and *V. alginolyticus* [7]. These species have also been reported to be associated with the HABs events [5,6], which could increase health risk and eco-hazards in marine environments. Consequently, efficient solutions that can mitigate both harmful phytoplankton and associated bacteria are encouraged.

HABs are being reported in both freshwater and marine ecosystems. One common impact that is related is that these water masses can be used as a water source for the raw influent of drinking water treatment plants (DWTPs) [9]. For instance, some DWTPs use carbon adsorption or chlorination, among other processes; however, they are still not efficient in removing these large blooms or associated toxins [2]. Additionally, chlorination involves the potential generation of by-products that are associated with the high levels of algal organic matter in these challenging waters. Another example is the use of desalination processes, where seawater reverse osmosis is the leading technology for those purposes. In this regard, one of the major operational problems is the accumulation of organic matter together with fouling complications that can be exacerbated due to the HABs’ episodes in coastal areas [10].

In this scenario, pretreatment methods have gained attention over the last years. Common biocides such as chlorine, CuSO_4,_ or KMnO_4_ have been investigated [11]. However, some disadvantages are also reported that are related to the formation of taste and odor compounds, disinfection by-products, etc. Accordingly, the use of alternative oxidants is also encouraged. Hydrogen peroxide can be one of them since H_2_O_2_ naturally degrades itself in water and oxygen. In fact, it has emerged as an attractive and environmentally friendly chemical for the selective abatement of cyanobacterial blooms in freshwater lakes [9,12,13]. The application of H_2_O_2_ is of special interest for cyanobacteria, which are prokaryotic cells with poorly elaborated mechanisms for H_2_O_2_ detoxification [3], although various sensitivities have been found among different cyanobacterial species [13]. Recently, assessing H_2_O_2_ oxidative stress on marine microalgae species has been reported [14,15,16]. However, specific studies focusing on marine cyanobacteria are limited.

On the other hand, persulfate salts have recently received widespread attention for use in water treatment but have been less studied for inactivating harmful phytoplankton [17]. One of the primary advantages of S_2_O_8_^2−^ for their application in seawater is the degradation products, as increasing levels of sulfates would be inconsequential compared with the background levels in seawater [17,18].

These oxidants are also widely applied in what is known as Advanced Oxidation Processes (AOPs), for which strong radicals may be generated and can accelerate inactivation practices. A clear example is the combination of either H_2_O_2_ or S_2_O_8_^2−^ with transition metals, such as iron, where it can efficiently react with the oxidants involving the highly reactive hydroxyl or sulfate radicals according to Equations (1) and (2) [19,20].

Fe^2+^ + H_2_O_2_ → Fe^3+^ + OH^−^ + ^●^OH (1)

Fe^2+^ + S_2_O_8_^2−^ → Fe^3+^ + 2 SO_4_^●−^ (2)

The use of these oxidants in the abatement of both cyanobacteria and related toxins together with bacteria has shown promising results in freshwaters [2,21,22,23]; however, the seawater scenario is less studied [17]. These studies make use of moderately high iron concentrations [17,21,23], which is important to consider because of the ecological risk of the residual metal after treatment. Additionally, the behavior of both cyanobacteria and bacteria inactivation in the consortium is limited [13]. Accordingly, the main goal of this study is to assess the efficacy of two different oxidants, H_2_O_2_ or S_2_O_8_^2−^, for the treatment of algae-laden waters.

## 2. Materials and Methods

### 2.1. Target Microorganisms

As target microorganisms, the marine cyanobacterium *Anabaena* sp. (Strain: CCMM 01/0101, provided by the Marine Microalgal Culture Collection of the Institute of Marine Sciences of Andalusia, ICMAN-CSIC; Supplementary Materials Figure S1) has been selected as representative of cyanobacterial fraction based upon their occurrence in source water supplies, the availability of a monoalgal culture, and the ability to produce odorous or toxic metabolites [24,25]. In parallel, the marine pathogen *Vibrio alginolyticus* (CECT521T; ATCC 17749) has been selected as the associated bacterial fraction [5,6,26].

*Anabaena* sp. were cultured in ground saltwater from the University Campus of Puerto Real at the University of Cadiz (pH = 7.65; salinity = 35.8) and enriched with Guillard f/2 medium (Guillard and Ryther, 1962). For experiments with Fe(II), the same f/2 medium was used but without trace metals and the EDTA complexing agent in order to avoid interferences with experimentation by adding iron as a possible catalyst (See Section 2.2). Experimentation was carried out in conditions that were able to simulate the algal bloom in a water source, i.e., with an initial cellular density of approx. 10^5^–10^6^ cells·mL^−1^. All cultures were maintained in a culture chamber at 20 °C illuminated by two LED lamps (Phillips LED tube, 18 W, 1600 lm, cool daylight) that provide photosynthetically active radiation of 130 μeinstein m^−2^ s^−1^, with a 14:10 light:dark cycle.

The monitoring of the *Anabaena* sp. cultures was followed by means of absorbance at 680 nm (Jenway 7315) and fluorescence (Microplate Fluorescence Reader “Tecan infinite F200”; excitation wavelength: 370 nm; emission wavelength: 670). Both measurements were correlated with cell concentration measured by microscopy (Leica, DM 750). Accordingly, the fluorescence or absorbance measurements and their corresponding values of cell concentration measured with Neubauer plates were subjected to a linear regression analysis. The intercept was not significant (*p* = 0.2380 and *p* = 0.8186 for absorbance and fluorescence, respectively), thus the cell density was linearly correlated with absorbance and fluorescence measurements with a slope of 2 × 10^−7^ (R^2^ = 0.7579) and 6·10^−4^ (R^2^ = 0.8591), respectively.

Alternatively, *V. alginolyticus* was cultured in marine broth (Panreac) and inoculated in either ground saltwater or cyanobacterial cultures to obtain a concentration of approx. 10^5^ CFU·mL^−1^. The reactivation of cryovials was performed as detailed elsewhere [27,28]. Bacterial survival after treatment was assessed with standard plate counts with the TCBS Agar (VWR Chemicals) [27].

### 2.2. Experimental Approach

Several experiments were performed with H_2_O_2_ (Scharlab 30% *w*/*v*) and peroxydisulfate salt (PDS), S_2_O_8_^2−^ (AppliChem 98%), which were applied in the range of 0.015–0.29 mmol/L and 0.05–5 mmol/L for H_2_O_2_ and S_2_O_8_^2−^, respectively. In this first step, the authors aim to determine the effect of both oxidants on *Anabaena* sp. on its own. Thus, growth inhibition tests were performed up to seven days in order to determine the effective values for 50% inhibition after 72 h but also the regrowth capability at longer times, which the authors consider of interest based on the aim of the present work.

Once the effect on *Anabaena* sp. caused by the addition of a single oxidant was determined, the possible enhancement of growth inhibition by the addition of low amounts (18 µmol/L) of Fe(II) (from FeSO_4_ (99%, Scharlab)) was studied in order to promote the generation of hydroxyl (^●^OH) or sulfate radicals (SO_4_^●−^) from H_2_O_2_ and S_2_O_8_^2−^, respectively (Equations (1) and (2)). In that case, selected concentrations of H_2_O_2_ and S_2_O_8_^2−^ based on the first experimental results were used, and similar growth inhibition tests were performed with the objective of observing if the presence of iron can increase the growth inhibition of cyanobacteria.

Finally, the efficiency of both oxidants together with the presence/absence of iron was assessed in co-cultures of *Anabaena* sp. and *Vibrio alginolyticus* as a consortium of cyanobacterial and marine bacteria. This was carried out to determine if the presence of bacteria can alter the results obtained from single cultures of cyanobacteria. The summarized experimental design is depicted in Table 1.

For *Anabaena* sp., a period of 6–9 days has been defined to determine the treatment effect. In the case of the bacterial assays (*V. alginolyticus*), the exposure time has been fixed for 72 h, according to the specific bacterial growth rates. All experiments were conducted in duplicate at least. Each chemical was added in a single dosage to reach the desired concentration in a total volume of 50 mL of culture. An aliquot of each culture was extracted daily to determine cell density (by means of absorbance or fluorescence measurement), together with oxidant decay and dissolved iron in selected cases. Dissolved iron, H_2_O_2_ and S_2_O_8_^2−^ were monitored spectrophotometrically according to the methods explained in Spuhler et al. 2010 [29], Eisenberg 1943, DIN 38 409 H15 [30]; and Liang et al. 2008 [31]. In all cases, the maximum volume to be extracted from each treated sample was limited to half of the original volume in order to avoid possible effects due to the loss of volume.

### 2.3. Data Treatment

In the case of cyanobacterial cells (*Anabaena* sp.), the effects from the different treatments were assessed by growth monitoring after the treatment. The growth inhibition (%) was calculated by the variation of cell density before and after treatment on each sampling day, as explained elsewhere [32]. For determining effective concentrations (EC50%) of specific reagents (H_2_O_2_ and S_2_O_8_^2−^), the growth inhibition (%) versus effective concentrations were fitted according to the model proposed by Hampel et al., 2001 [33]. Thus, the EC_50_% ± the standard error was obtained as a significant coefficient (*p* < 0.001) in the model.

In the case of bacteria (*V. alginolyticus*), the effects from the different treatments were assessed by analyzing inactivation profiles with a logarithmic reduction in the survival microorganisms (Log (N/N_0_)) versus time. The detection limit was determined as 10 CFU·mL^−1^, which corresponds to a 4.53–5.03 Log Removal Values (LRV).

## 3. Results and Discussion

### 3.1. Effects of H_2_O_2_ or S_2_O_8_^2−^ on Anabaena sp.

Firstly, in order to determine the damage that both oxidants can cause in *Anabaena* sp., H_2_O_2_ and S_2_O_8_^2−^ were applied in a wide range of doses to determine an effective concentration for both reagents. Although absorbance and fluorescence were valid for measuring cell concentration, the use of the fluorescence was selected due to its better correlation and higher sensitivity, which was essential in some cases when the concentration of the cultures is rather low. Additionally, the associated errors between replicates were reduced by means of fluorescence measurements (Supplementary Materials Figure S2).

**Figure 1:**
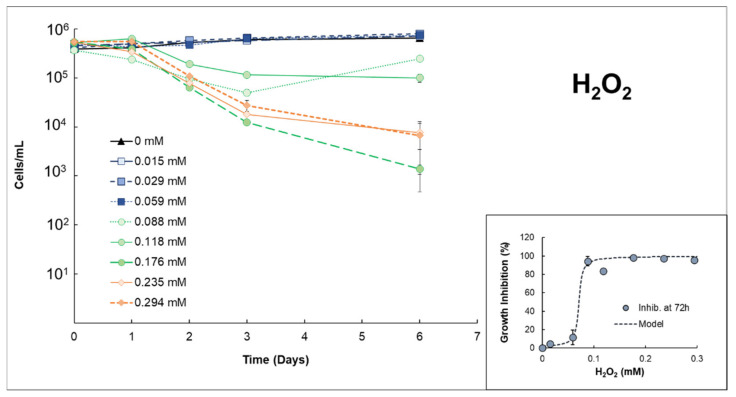
Growth curves of *Anabaena* sp. after adding different concentrations of H_2_O_2_. Inset: Growth inhibition rate at 72 h for *Anabaena* sp. exposed to H_2_O_2_. The line plotted corresponds to the fit of the model used (see Section 2.3. Data treatment).

In the case of H_2_O_2_ (Figure 1), low amounts were required to reach an effective concentration, EC50%, at 72 h = 0.0712 mmol/L ± 0.007 (R^2^ = 0.9845). In fact, concentrations equal to or higher than 0.088 mmol/L caused an inhibition percentage >90% (at 72 h) with respect to the control samples. This effect is somewhat modified throughout time since the EC50% on day 6 slightly increased up to 0.088 mmol/L ± 0.534 (R^2^ = 0.9628), suggesting that the damage cannot be reparable during this time frame at concentrations ≥0.088 mmol/L (2.99 mg H_2_O_2_/L).

**Figure 2:**
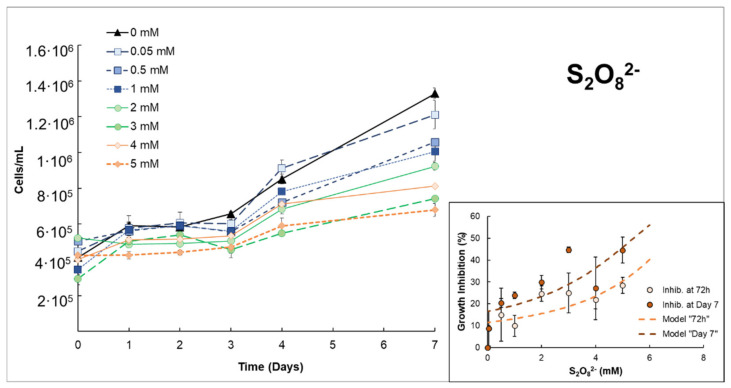
Growth curves of *Anabaena* sp. after adding different concentrations of S_2_O_8_^2−^ (PDS). Inset: Growth inhibition rate for *Anabaena* sp. exposed to S_2_O_8_^2−^ on specific days 3 and 7. The plotted line corresponds to the fit of the model that was used (see Section 2.3. Data treatment).

On the other hand, S_2_O_8_^2−^ (Figure 2) needs higher concentrations to cause perceptible cell damage since inhibition percentages of 8.71–28.40% were reached at 72h with oxidant doses of 0.5–5 mmol/L of S_2_O_8_^2−^. The estimated EC50% values at 72 h were obtained as 6.80 mmol/L ± 1.33 (R^2^ = 0.6678). In this case, those values were reduced up to 5.40 mmol/L ± 1.04 (R^2^ = 0.6878) on day 7, which suggests that the S_2_O_8_^2−^ react slowly with cells. Thus, the effects associated with S_2_O_8_^2−^ became perceptible at longer times. Nonetheless, the inhibition percentage is notably less if compared with the H_2_O_2_.

The consumption of oxidants was monitored during experimentation. In this context, differences were also evidenced according to the type of oxidant. When H_2_O_2_ was applied, rapid and total consumption (<24 h) was recorded for initial concentrations up to 0.18 mmol/L, whereas at least 72 h was necessary for total H_2_O_2_ consumption in the range 0.23–029 mmol/L. In respect to the PDS, longer consumption periods were detected. PDS consumption was quantified in the range of 25–55% after 7 days of exposure. These slow PDS consumption percentages can be related to the higher concentrations that were used but also to the higher stability of this salt. These results suggest the major efficiency of H_2_O_2_ over that of S_2_O_8_^2−^ when it is applied as a single oxidant, especially in cyanobacterial species.

Generally, hydrogen peroxide has shown significant sensitivity to cyanobacterial species with very low concentrations (2 mg H_2_O_2_/L; 0.059 mmol/L) [12,34]. These results corroborate the authors’ experiments with *Anabaena* sp., for which the EC50% values obtained (0.0712 mmol/L ± 0.007) agree with those reported in the literature with other cyanobacterial species, such as *Microcystis aeruginosa* [35,36]. These matches are interesting in the way *Anabaena* sp. differs from *M. aeruginosa* in cell morphology (filaments formed in *Anabaena* cultures) and also the seawater matrix used in this study.

The application of H_2_O_2_ for inactivating cyanobacteria in freshwater ecosystems was successfully applied in real blooms [3,12]. Thus, the specific sensitivity to H_2_O_2_ for the cyanobacterial species over that of other eukaryotic organisms was reported, showing that cyanobacterial species are more sensitive than other species of green algae or diatoms that show greater resistance to hydrogen peroxide [12,13,15,16,34]. The higher sensitivity of cyanobacteria to H_2_O_2_ can be related to the lack of major antioxidant enzymes, such as catalases or ascorbate peroxidase, which permits the degradation of substantial quantities of intracellular H_2_O_2_ [36,37]. The lack of these enzymes can be attributed to the fact that cyanobacteria do not have to deal with similar levels of intracellular H_2_O_2_ as do other eukaryotic microorganisms; therefore, cyanobacteria have less elaborate H_2_O_2_ detoxification routes [3,36,38].

On the other hand, the use of PDS salt (S_2_O_8_^2−^) results in low growth inhibition for *Anabaena* sp. even at higher concentrations of 5 mmol/L. The results in this study agree with previous studies in which the biocide efficacy of PDS was tested against natural groundwater microalgae [39] or green alga *Dunaliella tertiolecta* [17]. Results from these studies reported low biocidal activity of PDS. The results obtained in the present study suggests a minimum effect of PDS against cyanobacteria. Although some studies suggest possible intracellular damage provoked by the penetration of sulfate (that is in excess in the extracellular environment from S_2_O_8_^2−^) through the sulfate permeases (membrane-protein transporters) [40,41], it appears to be minimal. Indeed, the low consumption rates obtained suggest a slow reaction rate of PDS in seawater, supporting that PDS presents high stability in seawater [17].

### 3.2. Inactivation by the Presence of Fe (II)

As both H_2_O_2_ and S_2_O_8_^2−^ can be activated by the presence of transition metals, it seems interesting to know if the presence of small amounts of iron can be enough to increase the growth inhibition on *Anabaena* sp. For that purpose, selected concentrations of 0.059, 0.118 mmol/L of H_2_O_2_, and 2,3,4 mmol/L of S_2_O_8_^2−^ were combined by the addition of 18 µmol/L of Fe(II). Control tests with the single addition of iron were performed, and similar growth curves were observed as those without Fe(II), assuring that the addition of this metal in tested concentrations does not inhibit the growth of *Anabaena* sp. This accords with studies that are more specific, which suggests that the dominance of *Anabaena azotica* was between 18 and 36 µmol Fe/L rather than at other Fe concentrations [42].

**Figure 3:**
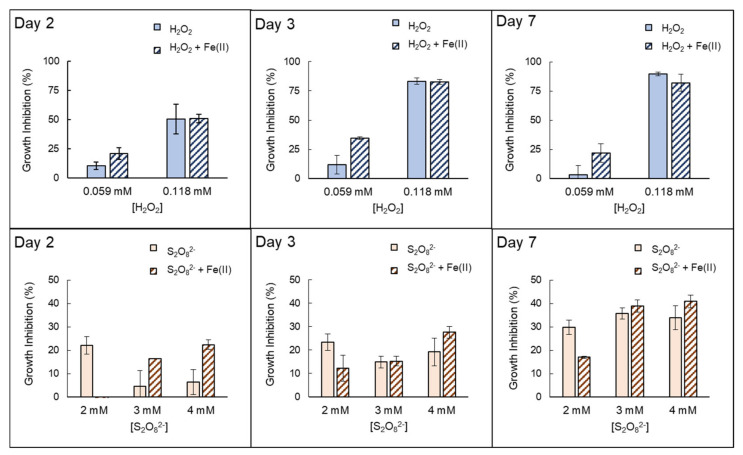
Specific growth inhibition (%) obtained at days 2, 3 and 7 for *Anabaena* sp. exposed to H_2_O_2_ or S_2_O_8_^2−^ in the presence or absence of Fe(II).

The inhibition growth (%) obtained for specific days 2, 3, and 7 are shown in Figure 3. In the case of H_2_O_2_, two different scenarios were observed. When concentrations of H_2_O_2_ were rather low (0.059 mmol/L), little effect in *Anabaena* sp. is observed (approx. 11% of growth inhibition on days 2 and 3), which becomes minimal on day 7 (3.17%), suggesting a recovery of these cells. In this case, ([H_2_O_2_] = 0.059 mmol/L), the combination with Fe(II) results in extra damage by obtaining an inhibition percentage of approximately 30% in respect to the control samples, which was maintained during the seven days of experimentation. Nonetheless, although higher growth inhibition was observed with the presence of iron in respect to single H_2_O_2_, this is still low for a possible abatement of the *Anabaena* blooms. Higher concentrations of H_2_O_2_ (0.118 mmol/L) imply a notably greater effect on the inhibition of growth, especially from day 3 onwards (> 80%, Figure 1 and Figure 3). These higher growth inhibition percentages deter from properly quantifying possible extra damage caused by the presence of iron, for which similar growth inhibition percentages were obtained (Figure 3).

When PDS (S_2_O_8_^2−^) is assessed as a source of radicals (Equation (2)), a low-moderate effect of S_2_O_8_^2−^ itself in *Anabaena* sp. was observed, which did not exceed 36% after seven days of experimentation (Figure 3). The addition of Fe(II) notably increased the effect of single PDS, for which the growth inhibition was enhanced by a factor of ~3.5 with 3 and 4 mmol/L of PDS on day 2. However, this improvement was decreasing with longer exposure time, where the increase in growth inhibition was by a factor of ~1.20 on day 7 for 3 and 4 mmol/L of PDS + Fe(II).

The addition of Fe(II) was spiking (in a single dosage) to the target cultures; hence, the presence of Fe(II) in the extracellular environment might lead to reactive radicals according to Equations (1) and (2), which can be responsible for cell damage in the bulk. However, the saline matrix, together with the basic pH of the *Anabaena* cultures, was probably responsible for the rapid oxidation of Fe(II) into Fe(III), decreasing the reaction rate among Fe(III) and H_2_O_2_ or S_2_O_8_^2−^ [19,43]. In fact, in the case of PDS, the reaction with Fe(III) is unknown [20].

Additionally, it would also involve the precipitation of iron hydroxides. In this regard, occasional measurements of dissolved iron were performed, which decreased up to 0.33–0.42 mg Fe/L within the first 24 h. It might entail heterogeneous Fenton-like reactions in the bulk to some extent and especially in the case of H_2_O_2_ [22]. On the other hand, an extracellular iron reduction from Fe(III) to Fe(II) could also occur facilitated by a specific outer membrane transporter on the cell surface or siderophore or other DOM-mediated mechanisms in the cultures [44,45,46], which could maintain the residual levels of dissolved Fe detected. These possible pathways were perhaps responsible for the enhanced growth inhibition at longer exposure times, specially for S_2_O_8_^2−^ (Figure 3).

Intracellular mechanisms might have also been responsible for the growth inhibition of *Anabaena* sp. after the iron addition in the presence of H_2_O_2_ or S_2_O_8_^2−^. The existence of these reagents, together with Fe in bulk, might have been transported to the intracellular domain [22,43]. The presence of additional H_2_O_2_ at the intracellular level can be fatal for cyanobacteria due to the lack of scavenging enzymes, as discussed in the previous Section 3.1. Thus, similar growth inhibition percentages were obtained with the presence/absence of iron at an H_2_O_2_ concentration of 0.118 mmol/L. However, S_2_O_8_^2−^, together with extracellular added iron, could have interacted with membrane transporters, favoring diffusion through the cell wall membrane. These hypothetic intracellular S_2_O_8_^2−^, together with the presence of extra iron, could have promoted an enhanced intracellular PDS/Fe(II) process, causing the enhancement observed in Figure 3 [22,40].

Although some improvements for the use of H_2_O_2_ or S_2_O_8_^2−^ in combination with low amounts of Fe(II) were detected, there was still a wide range to obtain higher growth inhibition in *Anabaena* sp. (especially for the PDS case), such as the addition of other activation factors (e.g., UV-radiation) or by increasing the iron concentration [32].

### 3.3. Behavior of Anabaena sp. Inactivation in Consortium with V. alginolyticus

As a next step, mixture experiments were performed with *Anabaena* sp. and *V. alginolyticus*, a marine pathogenic bacterium that has been associated with HABs.

According to the previous results described in Section 3.1 and Section 3.2, 0.088 mmol/L of H_2_O_2_ and 3 mmol/L of PDS were selected as the oxidant concentration, together with 18 µmol/L of Fe(II). Control experiments for assuring the regular growth of both *Anabaena* sp. and *V. alginolyticus* in co-cultures were performed, showing no growth inhibition for neither *Anabaena* sp. nor *V. alginolyticus*.

**Figure 4:**
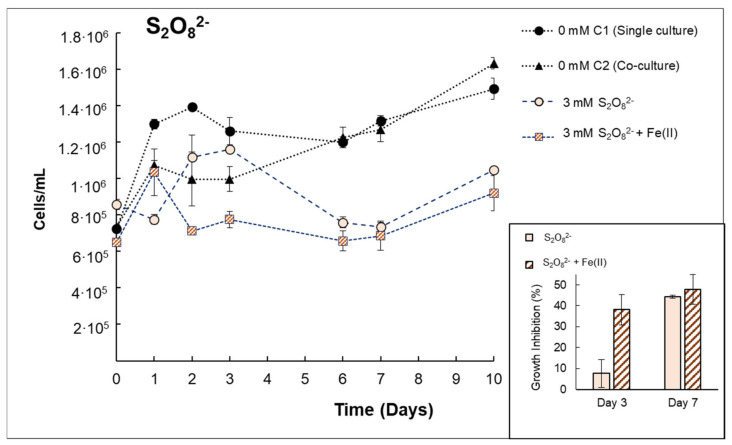
Growth curves of *Anabaena* sp. in co-culture with *V. alginolyticus* for single S_2_O_8_^2−^ or S_2_O_8_^2−^ + Fe(II) treatments. Inset: Specific growth inhibition (%) obtained on day 3 or 7 for *Anabaena* sp.

Regarding the use of PDS (Figure 4), similar trends were observed for *Anabaena sp.* when PDS was tested in monocultures. Some differences in growth inhibition were detected within the first days of exposure among PDS or PDS/Fe(II); however, similar growth inhibition percentages were observed on day 7 (44.29–47.73%). This suggests that similar damage was caused by these reagents in the presence of bacteria.

**Figure 5:**
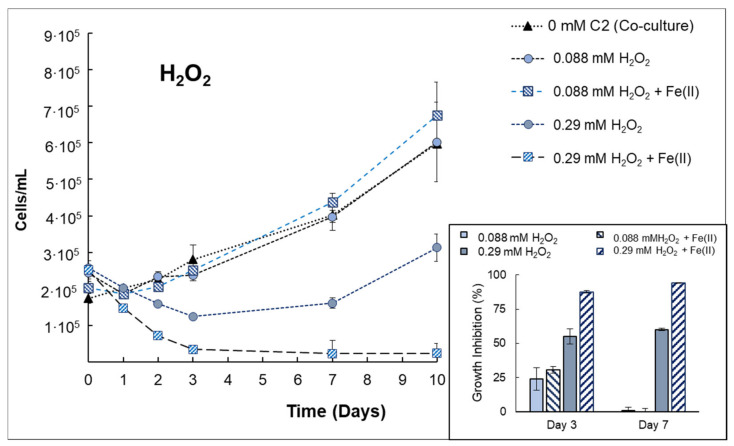
Growth curves of *Anabaena sp.* in co-culture with *V. alginolyticus* for single H*_2_*O*_2_* or H*_2_*O*_2_* + Fe(II) treatments. Inset: Specific growth inhibition (%) obtained at day 3 or 7 for *Anabaena* sp. exposed to H*_2_*O*_2_* or H*_2_*O*_2_* + Fe(II) treatments.

Experiments with H_2_O_2_ are depicted in Figure 5. Initially, a concentration of 0.088 mmol/L of H_2_O_2_ was used, for which growth inhibition was expected to some extent (see Section 3.1). However, with the presence of *V. alginolyticus,* the inhibition growth obtained for *Anabaena* sp. was quantified rather low, i.e., 23.91% and 30.57% (on Day 3) for both H_2_O_2_ and H_2_O_2_/Fe(II) treatments, respectively. The obtained results differ from those obtained by single *Anabaena* sp. cultures (Figure 1) as *Anabaena* sp. (in co-culture) is able to grow similar to control samples on day 7 (Figure 5). Accordingly, the authors decided to increase the concentration of H_2_O_2_ up to 0.29 mmol/L of H_2_O_2_, for which complete inhibition was observed in monocultures. Interestingly, the growth inhibition now decreased down to 60% on day 7 with the presence of *V. alginolyticus* (compared to the 99.01% obtained in monocultures, Figure 1). In addition, the differences were now very clear among H_2_O_2_ and H_2_O_2_/Fe(II) treatments where the inhibition percentage of *Anabaena* sp. reached 94% on day 7 with 0.29 mmol/L of H_2_O_2_ +Fe(II).

**Figure 6:**
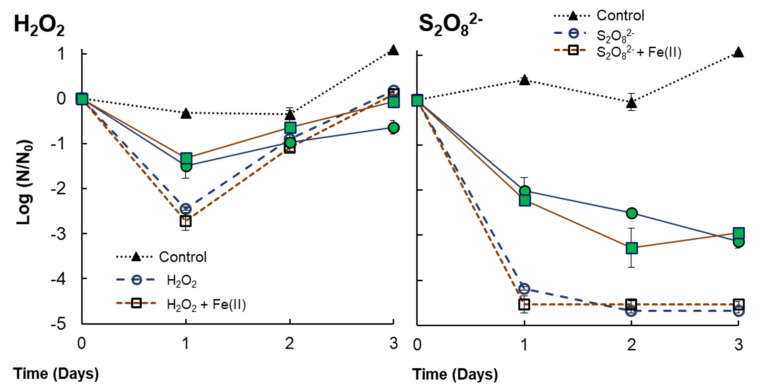
Inactivation of *V. alginolyticus* for single H*_2_*O*_2_* or S_2_O_8_^2−^ (+ Fe(II)) treatments [H*_2_*O*_2_*] = 0.29 mmol/L; [S_2_O_8_^2−^] = 3 mmol/L; [Fe(II)]= 18µmol/L. Filled markers indicate co-cultures with *Anabaena* sp., while empty markers indicate single cultures of *V. alginolyticus*.

Related to *V. alginolyticus*, no differences were detected by PDS or PDS/Fe(II) (Figure 6), although a delayed inactivation was observed for *V. alginolyticus* with the presence of *Anabaena* sp. It is important to note that PDS was not entirely consumed during experimentation, i.e., on day 7; 79.3% and 92% of the initial PDS amounts (3 mmol/L) were consumed by PDS and PDS/Fe(II), respectively. This remaining oxidant could be one of the reasons why *V. alginolyticus* was not able to regrow after treatment. With respect to the effect of hydrogen peroxide on *V. alginolyticus* survival (Figure 6), a 1.31–1.48 LRV or 2.44–2.72 LRV was obtained (within 24 h) in co-cultures or monocultures, respectively. However, after 24 h, bacteria regrowth was observed in both cases, which was probably caused by the bacteria that survived. This observed phenomenon could be due to the fact that H_2_O_2_ was entirely consumed within the first 24 h of the experiment. It could have permitted the surviving bacteria to grow, which is contrary to what happens with PDS, for which the high concentrations involved a residual oxidant that might have avoided the regrowth of bacteria. Nonetheless, treated samples did not reach control samples, suggesting the growth rate slows down, especially when *V. alginolyticus* was in a co-culture with *Anabaena* sp. (Figure 6). It might imply that the remaining bacteria are somehow damaged by the addition of H*_2_*O*_2_* (+Fe(II)) or by cyanobacterial-derived organic matter that was probably released during the treatment, which can affect the growth of *V. alginolyticus* [26].

The results obtained in co-cultures of *Anabaena* sp. and *V. alginolyticus* indicate the protection of cyanobacteria (extremely sensitive to H_2_O_2_) by the presence of bacteria, such as an increment of H_2_O_2_ to reach a growth inhibition of 50% was needed from 0.071 mmol/L (2.41 mg H_2_O_2_/L) up to 0.29 mmol/L (10 mg H_2_O_2_/L) in monocultures and co-cultures, respectively. It suggests that *Anabaena* sp. can survive at much higher H_2_O_2_ concentrations in a co-culture with marine bacteria. This effect was also recently reported by Weenink et al. 2021 [47], who demonstrated that green algae (*Chlorella sorokiniana*) could protect cyanobacteria (*Microcystis aeruginosa*) against oxidative stress originated by H_2_O_2_. Similar experiments, but with different approaches, were performed by Pulgarin et al. 2020 [22], in which the photo-Fenton process was tested against fecal bacteria (*E. coli*) in microalgal cultures (*Chlorella vulgaris*), suggesting, in this case, some protective effect for *E. coli* in *C. vulgaris* cultures. Thus, the presence of bacteria or green algae, with much developed cellular defenses against Reactive Oxygen Species, would degrade H_2_O_2_ more efficiently and could protect cyanobacteria against oxidative stress.

In addition, the effect of the Fenton process has also been evidenced in co-cultures where an increase of 58.4% in growth inhibition (on day 7) was observed for H_2_O_2_/Fe(II) compared to single H_2_O_2_. As previously stated (see Section 3.2), the rapid oxidation of Fe(II) into Fe(III) involves the iron precipitation in co-cultures; thus, the concentration of dissolved iron decreased within 24 h. Consequently, the Fenton reaction in an extracellular environment is expected to be rather slow. However, it is known that internal Fenton reactions can also occur [48]. Taking into account that marine cyanobacteria have iron-rich photosynthetic machinery with an extensive range of iron stress responses (due to the iron limitation in marine environments) [43], intracellular processes might become important. These could be responsible for observed growth inhibition with the presence of additional iron in the bulk. In combination with H_2_O_2_ and the lack of specific enzymes that could degrade it, the internal Fenton processes would be favored for the cyanobacterial case.

## 4. Conclusions

In this study, the efficacy of two different oxidants, H_2_O_2_ and S_2_O_8_^2−^ (PDS), was assessed against *Anabaena* sp. as bloom-forming and noxious cyanobacteria. A summary of the key findings is reported in Table 2.

The effects of both oxidants differed when they were assessed in monocultures of *Anabaena* sp. since H_2_O_2_ shows greater efficiency over that of PDS. *Anabaena* sp. was very sensitive to H_2_O_2_ (EC_50_% at 72 h = 0.0712 mmol/L), while PDS showed a moderate effect on growth inhibition (EC_50_% at 72 h = 6.80 mmol/L). With respect to H_2_O_2_, the addition of Fe(II) at 18 µmol/L did not increase the growth inhibition of *Anabaena* sp. due to the substantial sensitivity of the H_2_O_2_ itself. On the other hand, the growth inhibition (on day 3) was increased by a factor of ~3.5 with 3 and 4 mmol/L of PDS. This enhancement disappears at longer exposure times, reaching inhibition percentages never higher than 50%.

By co-culturing *Anabaena* sp. and *V. alginolyticus,* an incremental addition of H_2_O_2_ (from 0.071 mmol/L to 0.29 mmol/L) was required to achieve growth inhibition percentages higher than 50%, indicating that the presence of bacteria can protect cyanobacteria from H_2_O_2_ exposure. In addition, the effect of H_2_O_2_+Fe(II) was evidenced in co-cultures, increasing the growth inhibition by 58.4% compared to single H_2_O_2_. However, these H_2_O_2_ concentrations were not enough to prevent the regrowth of *V. alginolyticus* after 24 h.

The results that were obtained demonstrated that oxidants such as H_2_O_2_ or PDS salts (S_2_O_8_^2−^) can be applied to reduce both cyanobacteria and associated pathogenic bacteria in marine waters. Although some improvements about the use of H_2_O_2_ or S_2_O_8_^2−^ in combination with Fe(II) have been detected, there is still a wide range to promote higher growth inhibition in *Anabaena* sp., especially for PDS, for which moderate effects have been obtained.

## Figures and Tables

**Table 1:** Experimental approach for assessing H_2_O_2_- or S_2_O_8_^2−^-based processes on *Anabaena* sp. and *V. alginolyticus* in a consortium.

Target Organism	Treatment	[H_2_O_2_] (mmol/L)	[S_2_O_8_^2−^] (mmol/L)
*Anabaena* sp.	Single oxidant	0.015–0.29	0.05–5
	+ Fe(II) [Fe(II)] = 18 µmol/L	0.059, 0.118 ^1^	1, 3, 5 ^1^
*Anabaena* sp. + *V. alginolyticus*	Single oxidant	0.088, 0.29 ^2^	3 ^2^
	+ Fe(II) [Fe(II)] = 18 µmol/L	0.088, 0.29 ^2^	3 ^2^
*V. alginolyticus*	Single Oxidant	0.29	3
	+ Fe (II) [Fe(II)] = 18 µmol/L	0.29	3

^1^ Reagent concentrations selected based on the growth inhibition percentages obtained in Section 3.1; ^2^ Reagent concentrations selected based on the growth inhibition percentages obtained in Section 3.2.

**Table 2:** Summary table with key findings related to the use of H_2_O_2_ or S_2_O_8_^2−^ for inactivate *Anabaena* sp. and *V. alginolyticus* in (co)-cultures.

Target Organism	Treatment	Key Findings
***Anabaena*** **sp.**	H_2_O_2_	High growth inhibition (EC_50_% 72h = 0.0712 mmol H_2_O_2_/L ± 0.007). The strong effects of H_2_O_2_ itself inhibit properly quantifying possible extra damage caused by the presence of Fe(II).
	S_2_O_8_^2−^	Low–moderate growth inhibition ((EC_50_% 72 h = 6.80 mmol S_2_O_8_^2−^/L ± 1.33)). The addition of Fe(II) notably increases growth inhibition in the first 48–72 h. However, this improvement decreased with longer exposure times, e.g., on day 7.
***Anabaena*sp.** (+*V. alginolyticus*)	H_2_O_2_	The presence of bacteria implies increasing the H_2_O_2_ concentration up to 0.29 mmol H_2_O_2_/L to obtain a growth inhibition (at 72 h) of 55% ± 5.73, indicating that the presence of bacteria can protect cyanobacteria from H_2_O_2_ exposure. The effect of H_2_O_2_ + Fe(II) was evidenced in co-cultures, increasing the growth inhibition by 58.4% compared to single H_2_O_2_.
	S_2_O_8_^2−^	Similar growth inhibition percentages were observed when S_2_O_8_^2−^ was tested in monocultures (44.29–47.73% at day 7). This suggests that similar damage was caused by S_2_O_8_^2−^ in the presence of bacteria.
***V. alginolyticus*** (+ *Anabaena* sp.)	H_2_O_2_	H_2_O_2_ exposure (0.29 mM) implies 2.44 LRV at 24 h. It is reduced to 1.48 LRV with the presence of *Anabaena* sp. The consumption of H_2_O_2_ in the first 24 h would imply bacterial regrowth after this time. The addition of Fe(II) does not reflect an improvement in bacteria inactivation.
	S_2_O_8_^2−^	S_2_O_8_^2−^ exposures (3 mM) implies 4.18 LRV at 24 h. It is reduced to 2.01 LRV with the presence of *Anabaena* sp. No differences were detected by PDS or PDS/Fe(II). Residual S_2_O_8_^2−^ might avoid bacterial regrowth.

## Data Availability

Not applicable.

## Supplementary Materials

The following are available online at https://www.mdpi.com/article/10.3390/microorganisms10040735/s1, Figure S1. Anabaena sp. (Strain: CCMM 01/0101, ICMAN-CSIC). Figure S2. Growth curves of *Anabaena* sp. after adding different concentrations of H_2_O_2_. A. Cell density obtained by means of fluorescence measurements. B. Cell density obtained by means of absorbance (λ = 680 nm). C. Growth inhibition rate for *Anabaena* sp. at 72 h exposed to H_2_O_2_ by means of fluorescence of absorbance measurements.
