# Supplementary material for: On the Efficacy of H2O2 or S2O82− at Promoting the Inactivation of a Consortium of Cyanobacteria and Bacteria in Algae-Laden Water
Source: Microorganisms. 2022 Mar 29;10(4):735. doi: 10.3390/microorganisms10040735 (PMC9024476; doi:10.3390/microorganisms10040735)
Supplement: Supplementary file 1 [file microorganisms-10-00735-s001.zip › microorganisms-1601014-supplementary.pdf]

microorganisms

MDPI

Article

On the efficacy of H_2O_2 or $S_2O_8^{2-}$ to promote the inactivation of a consortium of cyanobacteria and bacteria in algae-laden water

Javier Moreno-Andrés[1,] *, Ignacio Rivas-Zaballos[1], Asunción Acevedo-Merino[1] and Enrique Nebot[1]

[1] Department of Environmental Technologies, Faculty of Marine and Environmental Sciences. INMAR - Marine Research Institute, CEIMAR - International Campus of Excellence of the Sea. University of Cadiz. Spain

* Correspondence: javier.moreno@uca.es

SUPPLEMENTARY MATERIAL

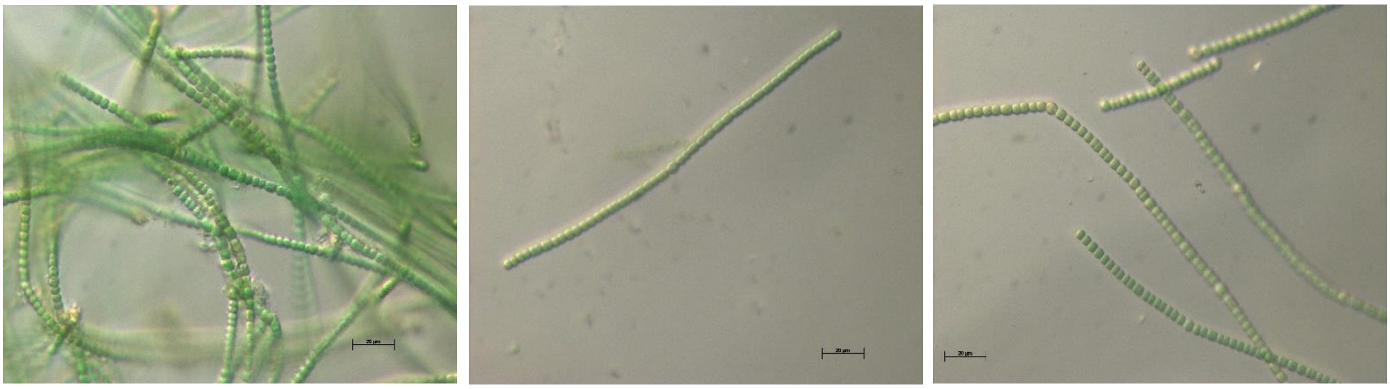

Figure S1. *Anabaena sp (*Strain: CCMM 01/0101, ICMAN-CSIC*).* The photographs have been taken with the help of Dr. Ignacio Moreno-Garrido, Department of Ecology and Coastal Management, Institute of Marine Sciences of Andalusia (ICMAN – CSIC).

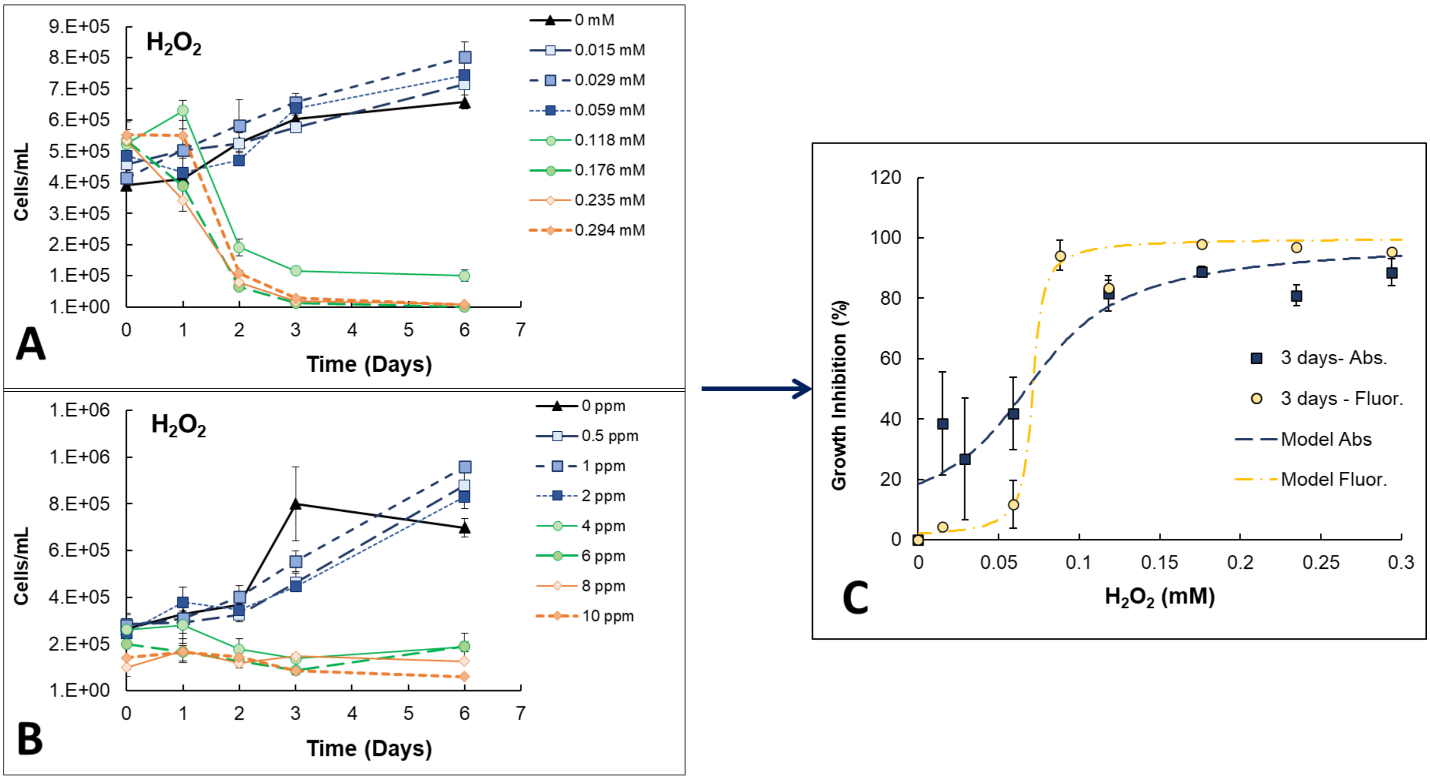

Figure S2. Growth curves of *Anabaena sp.* after adding different concentrations of H_2O_2. **A.** Cell density obtained by means of fluorescence measurements. **B.** Cell density obtained by means of absorbance (λ=680 nm). **C.** Growth inhibition rate for *Anabaena sp.* at 72 h exposed to H_2O_2 by means of fluorescence or absorbance measurements. The line plotted corresponds to the fit of the model used (see *2.3. Data treatment*).
